# Neonatal outcomes in the surgical management of placenta accreta spectrum disorders: a retrospective single-center observational study from 468 Vietnamese pregnancies beyond 28 weeks of gestation

**DOI:** 10.1186/s12884-024-06349-7

**Published:** 2024-04-02

**Authors:** Phuc Nhon Nguyen, Anh Dinh Bao Vuong, Xuan Trang Thi Pham

**Affiliations:** 1 Department of High-Risk Pregnancy, Tu Du Hospital, 284 Cong Quynh, Pham Ngu Lao Ward, District 1, Ho Chi Minh City, 71012 Vietnam; 2 Tu Du Clinical Research Unit (TD-CRU), Tu Du Hospital, Ho Chi Minh City, Vietnam

**Keywords:** Cesarean hysterectomy, MOSCUS, Emergency delivery, Placenta accreta spectrum disorders, Planned surgery, Neonatal outcome

## Abstract

**Background:**

Placenta accreta spectrum disorders (PASDs) increase the mortality rate for mothers and newborns over a decade. Thus, the purpose of the study is to evaluate the neonatal outcomes in emergency cesarean section (CS) and planned surgery as well as in Cesarean hysterectomy and the modified one-step conservative uterine surgery (MOSCUS). The secondary aim is to reveal the factors relating to poor neonatal outcomes.

**Methods:**

This was a single-center retrospective study conducted between 2019 and 2020 at Tu Du Hospital, in the southern region of Vietnam. A total of 497 pregnant women involved in PASDs beyond 28 weeks of gestation were enrolled. The clinical outcomes concerning gestational age, birth weight, APGAR score, neonatal intervention, neonatal intensive care unit (NICU) admission, and NICU length of stay (LOS) were compared between emergency and planned surgery, between the Cesarean hysterectomy and the MOSCUS. The univariate and multivariable logistic regression were used to assess the adverse neonatal outcomes.

**Results:**

Among 468 intraoperatively diagnosed PASD cases who underwent CS under general anesthesia, neonatal outcomes in the emergency CS (*n* = 65) were significantly poorer than in planned delivery (*n* = 403). Emergency CS increased the odds ratio (OR) for earlier gestational age, lower birthweight, lower APGAR score at 5 min, higher rate of neonatal intervention, NICU admission, and longer NICU LOS ≥ 7 days with OR, 95% confidence interval (CI) were 10.743 (5.675–20.338), 3.823 (2.197–6.651), 5.215 (2.277–11.942), 2.256 (1.318–3.861), 2.177 (1.262–3.756), 3.613 (2.052–6.363), and 2.298 (1.140–4.630), respectively, *p* < 0.05. Conversely, there was no statistically significant difference between the neonatal outcomes in Cesarean hysterectomy (*n* = 79) and the MOSCUS method (*n* = 217). Using the multivariable logistic regression, factors independently associated with the 5-min-APGAR score of less than 7 points were time duration from the skin incision to fetal delivery (min) and gestational age (week). One minute-decreased time duration from skin incision to fetal delivery contributed to reduce the risk of adverse neonatal outcome by 2.2% with adjusted OR, 95% CI: 0.978 (0.962–0.993), *p* = 0.006. Meanwhile, one week-decreased gestational age increased approximately two fold odds of the adverse neonatal outcome with adjusted OR, 95% CI: 1.983 (1.600–2.456), *p* < 0.0001.

**Conclusions:**

Among pregnancies with PASDs, the neonatal outcomes are worse in the emergency group compared to planned group of cesarean section. Additionally, the neonatal comorbidities in the conservative surgery using the MOSCUS method are similar to Cesarean hysterectomy. Time duration from the skin incision to fetal delivery and gestational age may be considered in PASD surgery. Further data is required to strengthen these findings.

**Supplementary Information:**

The online version contains supplementary material available at 10.1186/s12884-024-06349-7.

## Highlights

- The increased rate of poor neonatal outcomes in PASDs is mainly related to emergency cesarean section.

- The MOSCUS method and Cesarean hysterectomy are likely similar to the risk of adverse neonatal outcomes.

- Time duration from the skin incision to fetal delivery and gestational age are potential predictors in the surgery of PASDs.

**Supplementary Information:**

The online version contains supplementary material available at 10.1186/s12884-024-06349-7.

## Introduction

Placenta accreta spectrum disorders (PASDs), also called the abnormally invasive placenta, or morbidly adherent placenta is characterized by trophoblastic invasion into the myometrium. Depending on the depth of invaded tissue, PASDs includes the accreta, increta, and percreta. Globally, incidence is rapidly rising due to the increased rate of cesarean delivery [1]. However, PASDs can also appear on the uterus without a previous uterine scar [2]. Placenta accreta spectrum disorders cause a massive intraoperative hemorrhage which can lead to severe coagulopathy disorders, and hypovolemic shock and increase dramatically the rate of intensive care unit admission after cesarean section (CS) [3]. Thus, the morbidly adherent placenta remains a challenge for obstetricians and gynecologists which results in increasing significantly materno-fetal morbidity and mortality [4]. Proper management of PASDs including antenatal care and multidisciplinary care teams aim to improve the composite of maternal and neonatal morbidities and mortality, especially, in low-middle-income countries [5]. Despite an interdisciplinary approach, PASDs are associated with numerous complications with life-threatening conditions [6].

Routinely, almost all cases of abnormally invasive placenta in the second trimester are indicated for a termination of pregnancy [7]. The targeted delivery between 34 and 36 weeks of GA following types of PASDs was more likely associated with the improvement of maternal outcomes [8]. Meanwhile, expectation beyond 36 0/7 weeks of gestation is not advised since nearly one-half of women with placenta accreta spectrum beyond 36 weeks require emergency cesarean delivery for severe hemorrhage [9].

Several pieces of evidence have demonstrated that pregnancies along with PASDs are not associated with fetal growth restriction [10]. However, emergency cesarean surgery may relate to a lower birthweight due to premature delivery compared to planned cesarean surgery in the surgical management of PASDs [11]. Moreover, the poor neonatal outcome may be due to a lack of corticosteroids for fetal lung maturation in emergency delivery [12]. In a recently published report by Del Negro et al., PASD cesarean surgery has been significantly related to lower 5-min APGAR score, birthweight, and preterm gestational age compared to non-PASD cesarean section [13]. Therefore, antenatal diagnosis of PASDs is highly desirable. The materno-fetal outcomes are optimized when delivery occurs at specialist hospitals with expert obstetricians before the spontaneous onset of labor or catastrophic hemorrhage, and placental disruption [9].

Despite of paucity of evidence, the subsequent pregnancies of PASDs relate frequently to severe complications for both mother and fetus [14]. Thus, the neonatal outcomes in the present pregnancy with PASDs are a potential concern for all obstetricians worldwide, particularly, in low-resource settings. Appropriate timing of termination after considering the materno-fetal benefits is precisely required.

Previously, the PASDs are solely related to planned preterm Cesarean hysterectomy due to the high risk of postpartum hemorrhage [15]. Until today, many methods have been studied to reduce the massive blood loss in PASDs such as prophylactic placement of iliac balloons, Triple-P procedures, and conservative management [16]. Recently, conservative surgery can be accepted involving the development of an implemented interdisciplinary team. Therefore, the option of surgical methods should be individualized following the patient’s preference and available resources [17]. The modified one-step conservative uterine surgery (MOSCUS) is a novel approach to the management of PASDs at Tu Du Hospital, in Vietnam. This ongoing surgical experience has been applied since 2017, reducing blood loss substantially, and increasing the rate of uterine preservation [18–20]. To facilitate the surgery of PASDs, general anesthesia is potentially necessary. According to Ring et al., this intervention may cause fetal hypoxia, lower APGAR score at 5 min, respiratory distress syndrome requiring neonatal resuscitation, increased rate of assisted ventilation, and neonatal intensive care unit admission compared to the spinal epidural anesthesia. Since the fetus is vulnerable to maternal drug exposure during CS under general anesthesia [21]. Therefore, the neonatal outcome is a worrying issue in the context of PASDs surgery that needs a multidisciplinary team including an obstetrician, anesthetist, and neonatologist [22].

To our knowledge, a few papers mention the neonatal outcomes in emergency and planned cesarean surgery with the diverse surgical assessments among pregnant women with PASDs. Therefore, this study aimed to investigate and compare the perinatal outcomes between the emergency CS and planned CS as well as between the Cesarean hysterectomy and the MOSCUS method. Through this paper, we also sought to reveal some factors in predicting adverse neonatal outcomes in the clinical management of PASDs.

## Materials and methods

### Study design and participants

This was a secondary retrospective analysis of data from pregnant women with placenta accreta spectrum disorders (PASDs) who underwent cesarean delivery under general anesthesia applying standard procedure. The study was conducted between 2019 and 2020 at a single tertiary referral center in the south of Vietnam [20]. The pregnant women included were divided into two groups according to whether they had a planned or an emergency cesarean delivery. In addition, the surgical methods included conservative management using conventional assessment, the modified one-step conservative uterine surgery (MOSCUS), and Cesarean hysterectomy. STROBE guidelines were followed [23].

### The study criteria

#### Inclusion and exclusion criteria

**Fig. 1:**
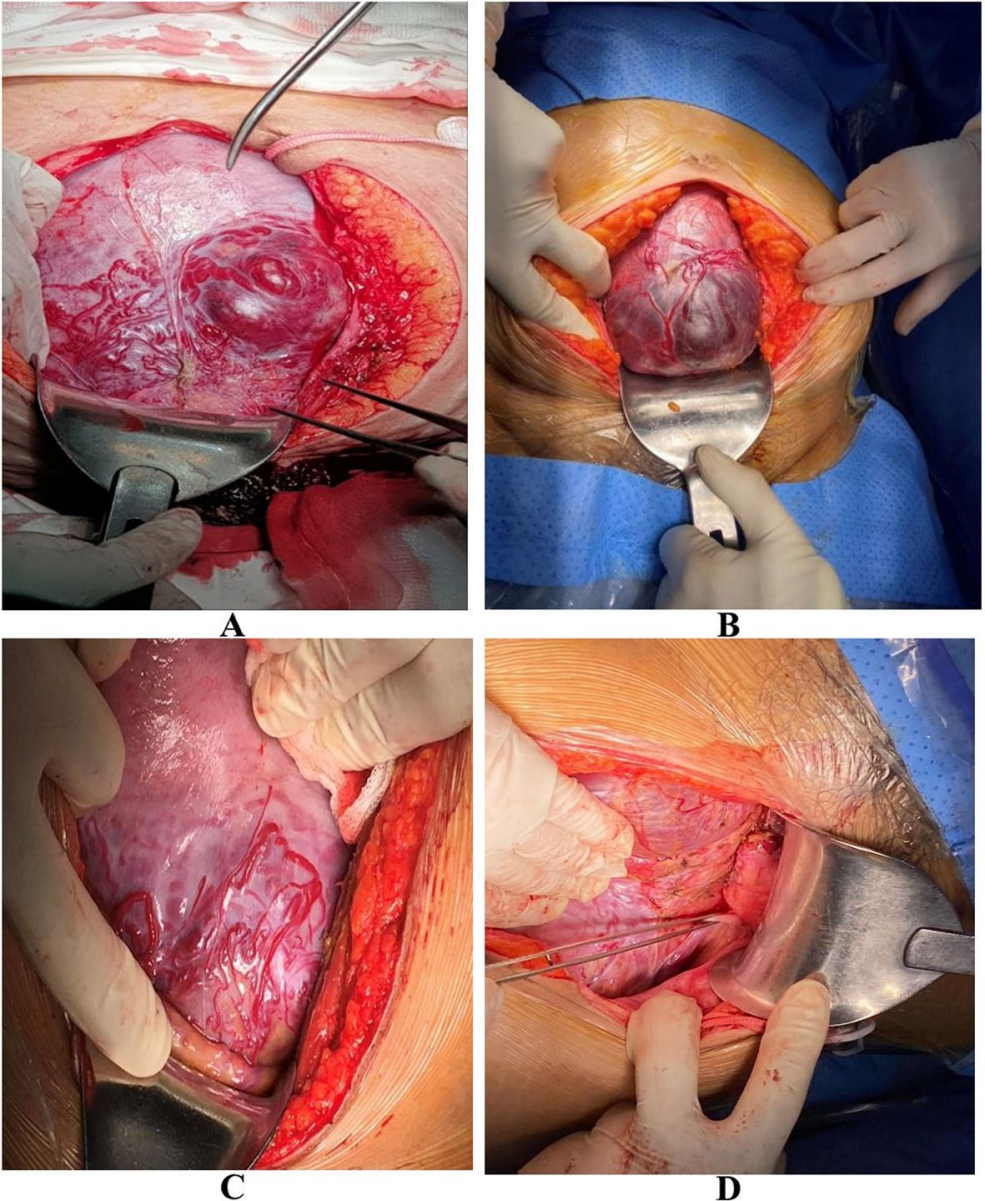
The appearances of placenta accreta spectrum disorders at laparotomy. The invaded placental budge can be clearly observed through a serosal layer (**A**-**B**). The uterine superficial surface before and after Step 1 (hemostatic procedures and vesicouterine dissection) of the MOSCUS method (**C**-**D**)

Singleton pregnancy beyond 28 weeks of gestation (gestational age of viability) were diagnosed with PASDs following the 10th revision of the International Classification of Diseases (ICD-10 code: O43) from the hospital medical record [24]. These patients underwent either emergency surgery or the planned surgery, as well as the Cesarean hysterectomy, and uterine conservative surgery with the MOSCUS method and without the MOSCUS method. At our center, the initial diagnosis of PASDs was assessed by antenatal sonography in the second trimester according to “International Federation of Gynecology and Obstetrics (FIGO) consensus guidelines on placenta accreta spectrum disorders (PASDs): Prenatal diagnosis and screening”. However, the accurate diagnosis was identified in surgery and by histopathology [25]. Exclusion criteria were (1) absence of intraoperative PASDs; (2) missed patient’s file; (3) absence of histopathological confirmation; (4) transferred hospital after undergoing cesarean section; (5) the neonatal death without identified etiology during the first 28 days of life; (6) termination of pregnancy due to maternal condition rather than that of PASDs pathology such as severe preeclampsia, and (7) fetal condition (severe congenital abnormalities, fetal distress) affecting to APGAR score rather than the general anesthesia. At the time of cesarean delivery, the exclusion of PASDs includes an absence of placental invasion on direct visualization, easy placental expulsion with synthetic oxytocin, and gently controlled cord traction. None of these features were considered for the diagnosis of PASDs [26]. Figure 1 shows typical PASD cases that were diagnosed in CS surgery.

To be confirmed with PASDs, a microscopic examination of the specimen originated from the whole uterus (in Cesarean hysterectomy) or the relevant tissue (uterine conservative surgery) must show extensive areas of absent decidua between villous tissue and the myometrium with placental villi attached directly to the superficial myometrium. The diagnosis cannot be made solely on gross observation of the delivered placental tissue or random biopsies of the placental bed. The range of pathologic adherence of the placenta determines the histologic classification of PASDs: accreta is limited to the decidual tissue layer, increta invades no further than the myometrial layer, and percreta extends into the serosal surface [26].

#### Emergency cesarean section and planned cesarean section

The emergency surgery was defined as the patient undergoing the cesarean section within 24 h after admission or the patient underwent CS before the scheduled date due to spontaneous labor (uncontrolled vaginal bleeding or persistent uterine contractions along with modification of the cervix). Whereas, the planned surgery also called “scheduled surgery” or “elective surgery” was identified beyond 24 h after hospitalization and programmed following the fixed date.

At our center, the timing of pregnancy termination could be individualized in cases of PASDs type of percreta with a severely invaded placenta into parametrium. After consideration of materno-fetal benefits with a multidisciplinary consultation, the termination of pregnancy at 32–34 weeks of gestation could be discussed with the patient and her family to mitigate the high risk of intraoperative complications. After obtaining the informed consent, the planned surgery of earlier gestational age could also be indicated to the patients who failed the tocolytic therapy using nifedipine or atosiban, repeated episodes of vaginal bleeding, and the patient was at risk of spontaneous labor at any moment.

The fetal lung maturation by administration of antenatal corticosteroid therapy was given at least 24–48 h before scheduled cesarean delivery.Pregnant women in emergency cesarean delivery were administered a single course of antenatal corticosteroid therapy for fetal lung maturity. The intravenous magnesium sulfate (MgSO4) for fetal neuroprotection was administered to the gestational age of 28–32 weeks within 24 h before delivery.

#### The modified one-step conservative uterine surgery (MOSCUS) and Cesarean hysterectomy

**Fig. 2:**
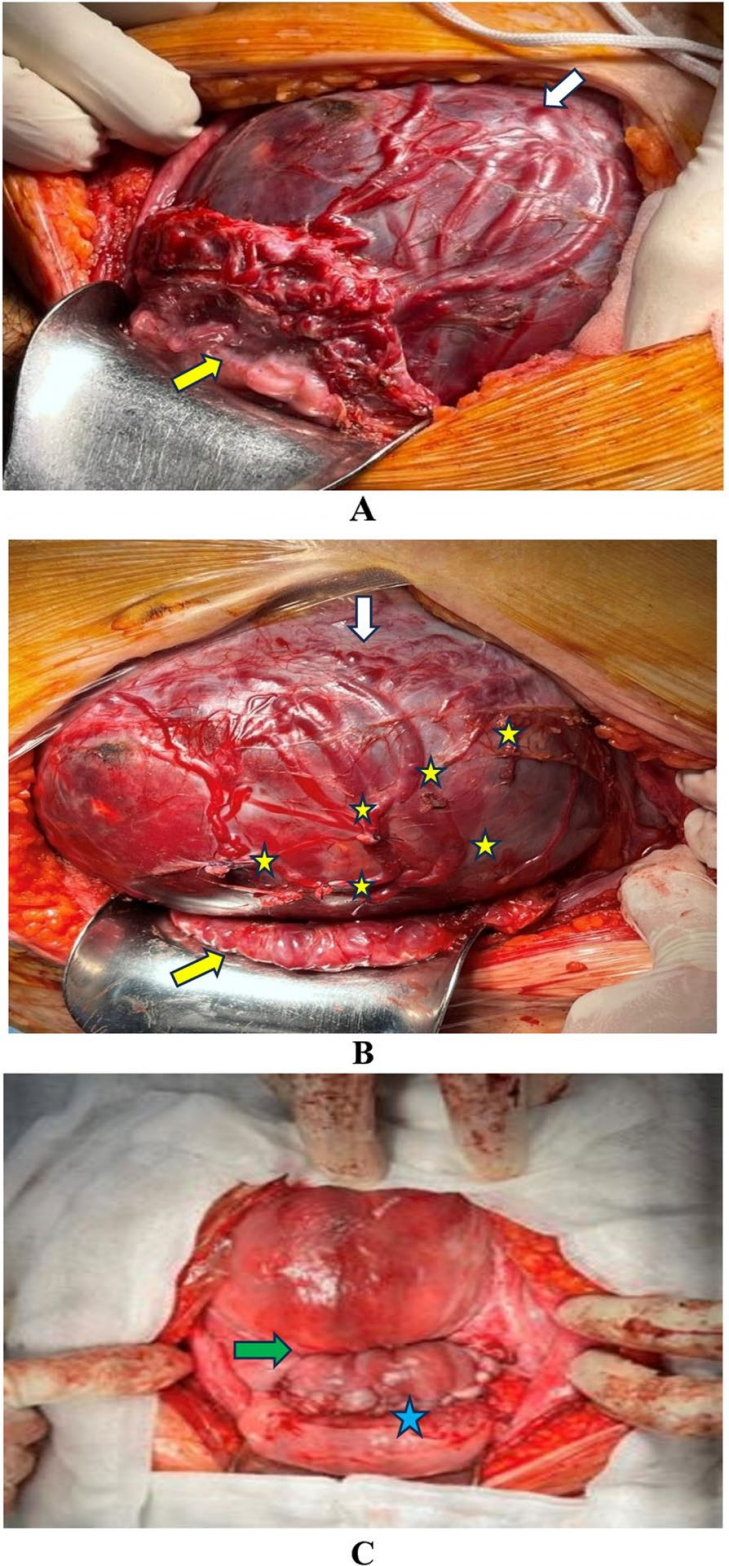
Photos show placenta accreta spectrum disorder type of percreta. **A** Site of uterine incision at the upper border of the placenta (white arrow), turbulent proliferative vascular at vesicouterine interface (yellow arrow). **B** Ligation of proliferative vascular in hemostatic procedure (yellow star) and the vesicouterine dissection is carefully performed, thus the bladder is lower than the initial location (yellow arrow)

The modified one-step conservative uterine surgery (MOSCUS) was a novel method in an assessment of PASD surgery at Tu Du Hospital, in Vietnam. The surgical method consists of five main steps, which purpose to avoid the long-term outcome of uterine removal and preserve female fertility [18, 20]. In five steps, the vesicouterine dissection along with hemostatic neovascular sutures before performing a uterine incision and fetal delivery (Step 1) (Supplementary Video 1–2) required meticulous performance. Step 1 also plays an important role in evaluating the lower uterine segment for conservative surgery (Fig. 2). Furthermore, this necessary step contributed to minimizing the estimated blood loss and prevented bladder perforation. Thus, this step could also be used in Cesarean hysterectomy. Cesarean hysterectomy was performed following the hospital protocol where applicable. All the remaining cases referred to conventional conservative surgery with hemostatic procedures such as placental bed suture, bilateral uterine artery ligation, CHO suture, Hayman suture, and B-Lynch uterine compression suture without sufficient steps described in MOSCUS methods. Delaying cord clamping for more than 30 s and skin-to-skin contact was not applied in CS surgery with PASDs since the patient underwent general anesthesia and the high risk of massive postpartum hemorrhage required immediate surgical procedures for hemostasis.

### Data collection

The continuous variables were maternal age (years), pre-pregnancy body mass index (kg/m^2^), hemoglobin level (g/dL), estimated blood loss (ml), time duration from skin incision to fetal extraction (min), total operative time duration (min), maternal length of stay in hospital (day), gestational age at birth (week), newborn birthweight (gram), APGAR score (point) at 1 min and 5 min, and length of stay in neonatal intensive care unit (NICU) (day). The categorical variables included parity, previous cesarean scar, maternal diseases, reason for admission, ureteral double-J insertion, mode of surgery (emergency CS/planned surgery), surgical method (the modified one-step conservative uterine surgery (MOSCUS) versus Cesarean hysterectomy), type of PASDs (accreta, increta, and percreta), placental site on ultrasound (anterior location/others), intraoperative complication, postoperative infection, requirement of blood transfusion, APGAR score (point) at 1 min (≤ 3 and > 3), at 5 min (< 7 and ≥ 7), ventilation support (supplementary oxygen, nasal positive pressure ventilation, or mechanical ventilatory assistance), and NICU admission. The neonatal outcomes were compared between emergency cesarean delivery and planned surgery as well as MOSCUS group and Cesarean hysterectomy. The *in-utero* fetal death cases before cesarean section under general anesthesia were considered for missing data of 1 min and 5 min- APGAR score. The multivariable analysis was carried out to reveal the factors relating to adverse neonatal outcome. Total score of APGAR at 5 min below 7 pts was considered an adverse neonatal outcome which required admission in neonatal intensive care unit (NICU), needing ventilatory support by continuous positive airway pressure (CPAP), invasive and non-invasive mechanical ventilation, and endotracheal intubation,…). Whereas, a total APGAR score of 8–10 pts was considered as good outcome. The newborn could stay with the mother on the first postoperative day if the maternal condition was appropriate (without NICU admission).

### Statistical analysis

Statistical analyses were performed using the Statistical Package for the Social Sciences (SPSS) version 26.0 (IBM Corp., Armonk, NY, USA). The analysis was performed using all available data, following the application of the exclusion criteria. The distribution of continuous variables was explored using histograms, skewness and kurtosis to identify a normal distribution. Descriptive statistics were expressed as mean and standard deviation (mean ± SD) for quantitative, percentages, and interquartile range for continuous variables depending on the distribution of data. The independent sample-t test and the Mann–Whitney U-test were used to compare continuous variables following the distribution of the data. Frequency data with percentage and comparison of categorical variables were performed across categories using the χ2 (chi-square test). If the counting variable has a theoretical number < 5 in each cell (> 25% of the table), the *p*-value is obtained by Fisher’s exact probability test. The odds ratio (OR) was calculated from the 2 × 2 Table. Indicators and effect values were expressed with a 95% confidence interval (CI). Univariate and multivariable logistic regression was calculated to identify the factors associated with poor neonatal outcomes and emergency cesarean section. The multivariable logistic regression was performed after including variables that had been identified as having a significant value (*p* < 0.05) in univariate analysis. Gestational age and birthweight were added to calculate the adjusted odds ratios (aOR) with 95% CI was provided. The goodness of fit was assessed by the Hosmer–Lemeshow test. A probability value of *p* < 0.05 was considered statistically significant for all analyses.

## Results

**Fig. 3:**
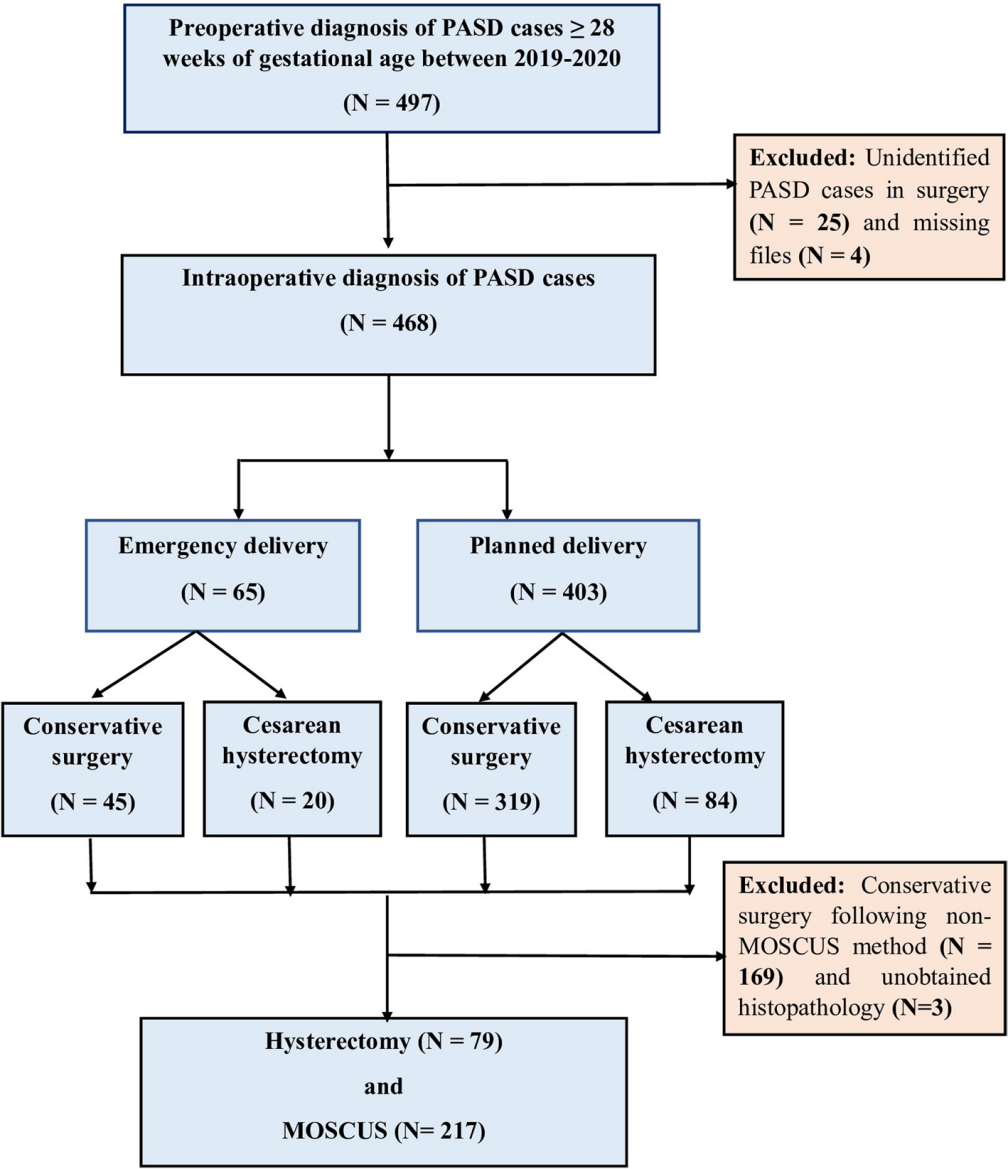
Study flowchart

In our cohort, a total of 468 pregnant women with placenta accreta spectrum disorders (PASDs) met inclusion criteria, 65 cases underwent emergency cesarean section (CS) delivery, and 403 cases underwent planned delivery. Out of these, the Cesarean hysterectomy was performed in 79 cases, and the modified one-step conservative uterine surgery (MOSCUS) was applied in 217 cases (Fig. 3). Among them, 240 cases were greater than 34 weeks of gestation. All the materno-fetal characteristics and surgical features are summarized in Table 1 and Supplementary Table 1.

**Table 1:** Baseline characteristics of study population

Characteristics			Emergency surgery *N* = 65	Planned surgery *N* = 403	Total *N* = 468	*p*-value
**Maternal age (years)**	mean ± SD		33.23 ± 4.52	33.25 ± 4.92	33.25 ± 4.87 (18–49)	0.973^*^
	≥ 35		27 (69.2)	166 (64.8)	193 (65.4)	0.592^┼^
	< 35		12 (30.8)	90 (35.2)	102 (34.6)	
**Pre-pregnancy BMI (kg/m**^**2**^**)**	mean ± SD		20.92 ± 2.47	21.87 ± 2.78	21.73 ± 2.75 (15.56 -33.29)	0.010^*^
**Parity (times)**	mean ± SD		1.65 ± 0.82	1.53 ± 3.22	1.54 ± 0.78 (0–6)	0.260^*^
	≥ 2		35 (53.8)	181 (44.9)	216 (46.2)	0.180^┼^
	< 2		30 (46.2)	222 (55.1)	252 (53.8)	
**Cesarean scar (times)**	≥ 2		25 (38.5)	124 (30.8)	149 (31.8)	0.217^┼^
	< 2		40 (61.5)	279 (69.2)	319 (68.2)	
**Maternal systemic diseases**	Healthy		30 (46.2)	229 (56.8)	259 (55.3)	0.056^┼^
	Anemia		22 (33.8)	108 (26.8)	130 (27.8)	
	Pre-eclampsia		0 (0.0)	11 (2.7)	11 (2.4)	
	Gestational diabetes mellitus		6 (9.2)	39 (9.7)	45 (9.6)	
	Others		7 (10.8)	16 (4.0)	21 (4.9)	
**Reason for admission**	Asymptomatic		9 (13.8)	281 (69.7)	290 (62.0)	** < 0.0001**^**┼**^
	Abdominal pain		6 (9.2)	31 (7.7)	37 (7.9)	
	Vaginal bleeding		50 (76.9)	91 (22.6)	141 (30.1)	
**Hb at admission (g/dL)**	mean ± SD		10.77 ± 1.72	11.43 ± 1.30	11.33 ± 1.39 (6.80–15.00)	0.004^*^
**Hb at pre-surgery (g/dL)**	mean ± SD		10.69 ± 1.75	11.47 ± 1.20	11.37 ± 1.31 (6.80–15.00)	0.001^*^
**Hb at discharge (g/dL)**	mean ± SD		8.91 ± 1.50	9.52 ± 1.56	9.44 ± 1.57 (5.50–14.40)	0.004^*^
**Ureteral double-J insertion**	No		56 (86.2)	180 (44.7)	236 (50.4)	** < 0.0001**^**┼**^
	Yes		9 (13.8)	223 (55.7)	232 (49.6)	
**Location of PASDs on US**	Absence of placenta previa		1 (1.5)	10 (2.5)	11 (2.4)	NA
	Anterior		31 (47.7)	188 (46.7)	219 (46.8)	
	Posterior		9 (13.8)	66 (16.4)	75 (16.0)	
	Right lateral		5 (7.7)	21 (5.2)	26 (5.6)	
	Left lateral		4 (4.6)	18 (4.5)	22 (4.7)	
	Middle		15 (23.1)	100 (24.8)	115 924.6)	
**Type of intraoperative PASDs**	Accreta		13 (20.0)	80 (19.9)	93 (19.9)	0.699^┼^
	Increta		24 (36.9)	129 (32.0)	153 (32.7)	
	Percreta		28 (43.1)	194 (48.1)	222 (47.4)	
**Time from skin incision to fetal delivery (mins)**	mean ± SD		21.76 ± 17.19	28.12 ± 16.19	27.23 ± 16.46 (2–95)	0.011^*^
**Operative time duration (mins)**	mean ± SD		133.00 ± 61.00	143.36 ± 50.00	141.92 ± 51.73 (50–365)	0.134^*^
**EBL (ml)**	median		1200	800	800	0.009^**^
	IQR [Q1-Q3]		[600–2450]	[500–1500]	[500–1600]	
	(min–max)		(200–5300)	(200–6500)	(200–6500)	
**Intraoperative complication**	Absence		61 (93.8)	380 (94.3)	441 (94.2)	NA
	Presence	Bladder perforation	4 (6.2)	16 (4.0)	20 (4.3)	
		Ureter injury	0 (0.0)	3 (0.7)	3 (0.6)	
		Rectal/bowel perforation	0 (0.0)	3 (0.7)	3 (0.6)	
		Large vessel injury	0 (0.0)	1 (0.2)	1 (0.2)	
**Hospital LOS (days)**	mean ± SD		7.20 ± 2.31	6.73 ± 2.06	6.80 ± 2.10 (2^a^-20)	0.098^*^
**Blood transfusion**	No		15 (23.1)	171 (42.4)	186 (39.7)	**0.003**^**┼**^
	Yes		50 (76.9)	232 (57.6)	282 (60.3)	
**Postpartum hemorrhage**	Absence		62 (95.4)	399 (99.3)	461 (98.7)	NA
	Presence	Medical therapy	2 (3.1)	1 (0.2)	3 (0.6)	
		Relaparotomy for hemostasis	0 (0.0)	1 (0.2)	1 (0.2)	
		Relaparotomy for secondary hysterectomy	1 (1.5)	1 (1.5)	2 (0.4)	
**Postoperative infection**	Absence		59 (90.8)	382 (95.0)	441 (94.4)	NA
	Presence	Medical therapy				
		Relaparotomy without hysterectomy	0 (0.0)	1 (0.2)	1 (0.2)	
		Relaparotomy with hysterectomy	0 (0.0)	1 (0.2)	1 (0.2)	

Statistically significant *p*-values (< 0 .05) are written in bold

*EBL* Estimated blood loss, *LOS* length of stay, *Hb* Hemoglobin, *US* Ultrasound

In 468 PASDs cases: In vitro fertility (*N* = 6), previous placenta previa in previous pregnancy (*N* = 22), uterine malformation (*N* = 2). In 434 PASDs cases with uterine scar: Cesarean scar (*N* = 431), myomectomy scar (*N* = 2), other (*N* = 1)

^a^One patient was recorded with 2-day- postoperative length of stay because the patients was transferred to a tertiary general hospital for peripartum cardiomyopathy

^*^Chi-squared test

┼Independent samples- t test, IQR [1–3]: interquartile range [25%-75%]

**Fig. 4:**
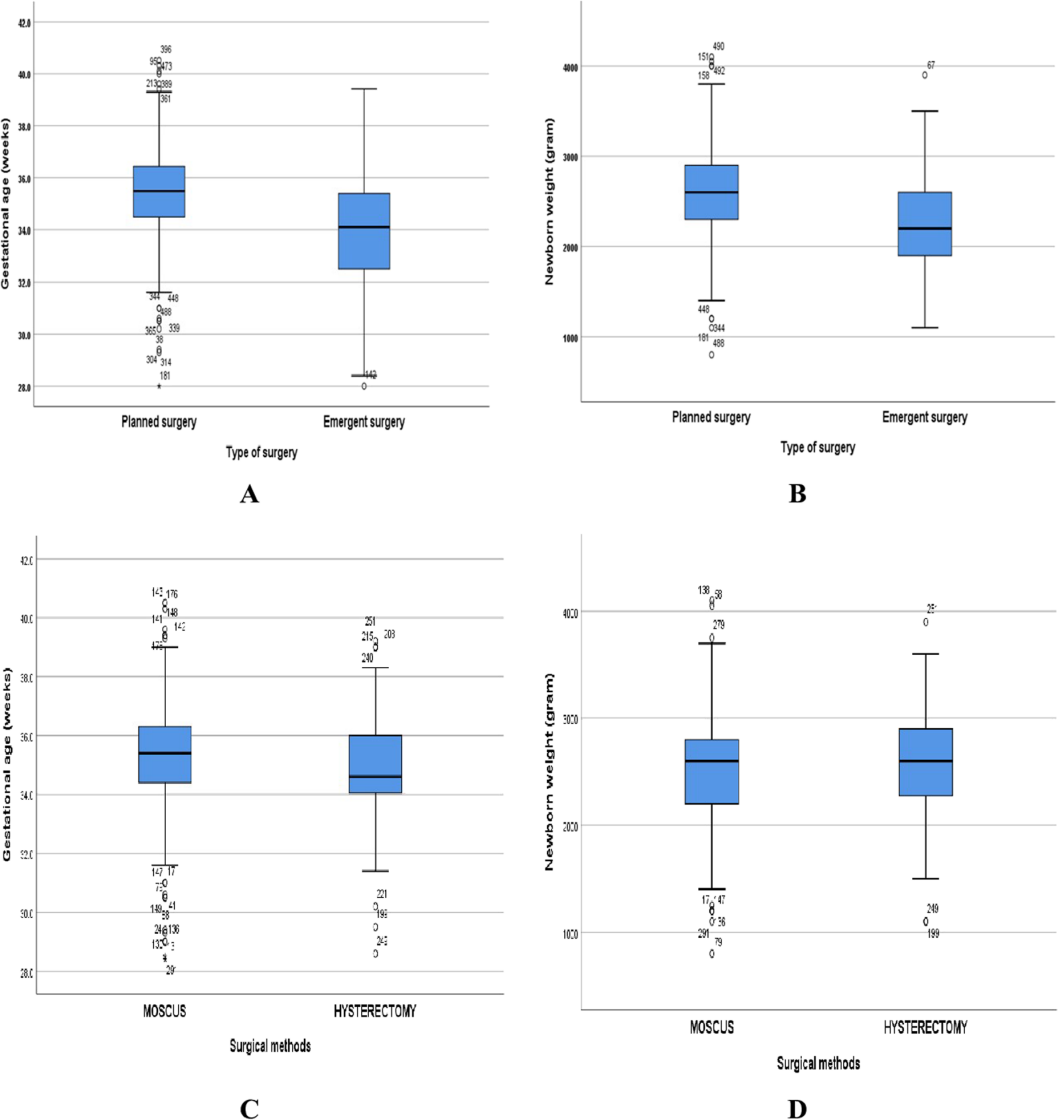
Box plots show: A. Mean gestational age (weeks) in PASDs following type of surgery. B. Mean newborn weight (gram) in PASDs following type of surgery. C. Mean gestational age (weeks) in PASDs following surgical methods. D. Mean newborn weight (gram) in PASDs following surgical methods

In this study, Table 2 shows there were no differences with statistical significance between emergency and planned CS surgery in some characteristics such as parity, the number of cesarean scars, maternal diseases, location of PASDs on ultrasound, mean operative time duration (min), hospital length of stay (LOS) (day), intraoperative and postoperative complications. However, some features were significantly different between the two groups including gestational age (week), birthweight (gram), reason for admission, hemoglobin concentration (g/dL), time duration from skin incision to fetal delivery (min), APGAR score at 1 min and 5 min, estimated blood loss (EBL) (ml), and blood transfusion (Fig. 4A-B and Supplementary Fig. 1A-B). All factors relating to emergency CS are presented in Supplementary Table 2.

**Table 2:** Neonatal outcomes of pregnancies underwent emergency surgery compared to planned surgery in the management of placenta accreta spectrum disorders

Neonatal outcomes		Emergency surgery *N* = 65		Planned surgery*N* = 403	Total *N* = 468	*OR 95% CI*	*p-value*
**Gestational age (wks)**	Mean (min–max)	34.02 ± 2.74		35.60 ± 1.63	35.38 (28–40.5)		< 0.0001┼
	< 34	27 (41.5)		25 (6.2)	52 (11.1)	**10.743 (5.675–20.338)**	< 0.0001*
	≥ 34	38 (58.5)		378 (93.8)	416 (88.9)	Ref	
**Birthweight (gr)**	Mean (min–max)	2203.08 ± 593.84		2624.30 ± 476.03	2565.67 ± 514.52 (800–4100)		** < 0.0001┼**
	< 2500	43 (66.2)		136 (33.8)	179 (38.3)	**3.823 (2.197–6.651)**	< 0.0001^*^
	≥ 2500	22 (33.8)		266 (66.2)	288 (61.7)	Ref	
**APGAR score 1 min (points)**	Median	5		5	5		0.004^**^
	IQR	[4–6]		[5, 6]	[5, 6]		
	[1–3] (min–max)	(1–7)		(1–7)	(1–7)		
	≤ 3	11 (16.9)		15 (3.8)	26 (5.6)	**5.215 (2.277–11.942)**	** < 0.0001**^**┼┼**^
	> 3	54 (83.1)		384 (96.2)	438 (94.4)	Ref	
**APGAR score 5 min (points)**	Median	7		7	7		0.002^**^
	IQR	[6, 7]		[6, 7]	[6, 7]		
	[1–3]	(1–9)		(1–8)	(1–9)		
	< 7	29 (44.6)		105 (26.3)	134 (28.9)	**2.256 (1.318–3.861)**	0.003*
	≥ 7	36 (55.4)		294 (73.7)	330 (71.1)	Ref	
**Requirement for oxygen support**	Yes	42 (64.6)		182 (45.6)	224 (48.3)	**2.177 (1.262–3.756)**	< 0.004^*^
	No (air oxygen)	23 (35.4)		217 (54.4)	240 (51.7)	Ref	
	Type of ventilation	Cannula/mask	22 (33.8)	134 (33.6)	156 (33.6)		
		CPAP	10 (15.4)	24 (6.0)	34 (7.3)		
		Intubation	10 (7.3)	24 (6.0)	34 (7.3)		
**Admission at NICU**^**a**^	Yes	44 (68.8)		151 (37.8)	195 (42.1)	**3.613 (2.052–6.363)**	< 0.0001^*^
	No	20 (31.2)		248 (62.2)	268 (57.9)	Ref	
**Length of stay at NICU (days)**	Median	8.5		6	7		0.002^**^
	IQR [1–3]	[6.0–14.75]		[6–9]	[5–11]		
	(min–max)	(3–51)		(1–56)	(1–56)		
	≥ 7	29 (65.9)		69 (45.7)	98 (50.3)	**2.298 (1.140–4.630)**	0.018^*^
	< 7	15 (34.1)		82 (54.3)	97 (49.7)	Ref	
**Major neonatal outcome**^**b**^	Death/Transferred hospital	0 (0.0)		6 (1.5)	6 (1.3)	NA	1.0^┼┼^
	Alive	65 (100.0)		396 (98.5)	461 (98.7)		

Statistically significant *p*-values (< 0 .05) are written in bold

IQR [1–3]: interquartile range [25%-75%]

^a^Four neonates were death and one was transferred to pediatric hospital without admission to NICU. Missing data was calculated for in-utero fetal death before cesarean section under general anesthesia

^b^Severe neonatal jaundice requiring phototherapy was recorded in 10 cases

^*^Chi-squared test

^**^Independent sample Mann Whitney test

┼Independent samples- t test,

┼┼Fisher’s exact test, *p*-value of exact sign (2-sided test)

Overall, the neonatal outcomes in the emergency CS surgery versus the planned CS surgery are shown in Table 2. Emergency delivery is related to the risk of neonatal complications consisting of gestational age less than 34 weeks of GA, lower birth weight, lower APGAR score at 1 min, 5 min, need for ventilatory support, the requirement for NICU admission, and LOS at NICU more than 7 days with OR (95% CI): 10.743 (5.675–20.338), 3.823 (2.197–6.651), 5.215 (2.277–11.942), 2.256 (1.318–3.861), 2.177 (1.262–3.756), 3.613 (2.052–6.363), and 2.298 (1.140–4.630), respectively, *p* < 0.05.

Meanwhile, Table 3 shows the neonatal outcomes in the MOSCUS method are similar to those of Cesarean hysterectomy, the difference was not statistically significant (Fig. 4C-D and Supplementary Fig. 1C-D).

**Table 3:** Neonatal outcomes in the management of placenta accreta spectrum disorders underwent MOSCUS group compared to Cesarean hysterectomy

Neonatal outcomes			MOSCUS *N* = 217	Hysterectomy*N* = 79	Total *N* = 296	*OR 95%CI*	*p*-value
**Gestational age (weeks)**	Mean ± SD (min–max)		35.32 ± 1.98	35.07 ± 1.97	35.25 ± 1.97 (28.4—40.5)		0.340^┼^
	< 34		26 (12.0)	8 (10.1)	34 (11.5)	1.208 (0.523–2.793)	0.658^*^
	≥ 34		191 (88.0)	71 (89.9)	262 (88.5)	Ref	
**Birth weight (gram)**	Mean ± SD (min–max)		2541.75 ± 527.00	2538.09 ± 498.27	2540.77 ± 518.65 (800–4100)		0.957^┼^
	< 2500		86 (39.6)	35 (44.3)	121 (40.9)	0.825 (0.490–1.389)	0.469^*^
	≥ 2500		131 (60.4)	44 (55.7)	175 (59.1)	Ref	
**1 min APGAR score (point)**	Median		5	5	5		0.747^┼^^┼^
	IQR [1–3]		[5, 6]	[5, 6]	[5, 6]		
	(min–max)		(1–7)	(1–7)	(1–7)		
	≤ 3		15 (7.0)	4 (5.1)	19 (6.5)	1.388 (0.446–4.316)	0.570^*^
	> 3		200 (93.0)	74 (94.9)	277 (93.6)	Ref	
**5 min APGAR score (points)**	Median		7	7	7		0.761^┼┼^
	IQR [1–3]		[6, 7]	[6, 7]	[6, 7]		
	(min–max)		(4–8)	(3–9)	(3–9)		
	< 7		73 (34.0)	25 (32.1)	98 (33.4)	1.090 (0.627–1.895)	0.760^*^
	≥ 7		142 (66.0)	53 (67.9)	195 (66.6)	Ref	
**Oxygen support requirement**	Yes		112 (51.6)	39 (49.4)	151 (51.0)	1.094 (0.654–1.831)	0.732^*^
	No		105 (48.4)	40 (50.6)	145 (49.0)	Ref	
	Type of ventilation	Cannula/mask	77 (35.5)	30 (38.0)	107 (36.1)		
		CPAP	17 (7.8)	4 (5.1)	21 (7.1)		
		Mechanical ventilation^a^	18 (8.3)	5 (6.3)	23 (7.8)		
**Admission at NICU**	Yes		94 (43.3)	36 (45.6)	130 (43.9)	0.913 (0.544–1.532)	0.73^*^
	No		123 (56.7)	43 (54.4)	166 (56.1)	Ref	
**Length of stay at NICU** **(days)**	Median		6	7	6		0.646^┼┼^
	IQR [1,–3]		[4,–10]	[4,–12]	[4,–10]		
	(min–max)		(1–41)	(1–50)	(1–50)		
	≥ 7		40 (42.6)	20 (55.6)	60 (46.2)	0.593 (0.273–1.285)	0.183^**^
	< 7		54 (57.4)	16 (44.4)	70 (53.8)	Ref	
**Major neonatal outcomes**	Death/Transferred hospital		4^b^ (1.8)	2^c^ (2.5)	6 (2.0)	0.723 (0.130–4.027)	0.659^**^
	Alive		213 (98.2)	77 (97.5)	290 (98.0)	Ref	

*CPAP* Continuous positive airway pressure, *NICU* Neonatal intensive care unit, *Hb* Hemoglobin

IQR [1–3]: interquartile range [25%-75%]

^a^Mechanical ventilation including invasive and non-invasive method

^b^intrauterine fetal demise (*n* = 1) and termination of pregnancy (*n* = 1), neonatal death due to severe pneumonia/respiratory distress syndrome (*n* = 1), and transferred hospital due to pulmonary effusion/pneumonia (*n* = 1)

^c^in-utero fetal death (*n* = 1), transferred hospital due to the severe respiratory distress syndrome (*n* = 1). Missing data was calculated for in-utero fetal death before cesarean section under general anesthesia

^*^Chi-square test

^**^Fisher’s exact test

^┼^Independent sample-t test

^┼┼^Independent sample Mann Whitney test, asymptotic sig (2-sided) of *p*-value

By further exploration of the adverse neonatal outcomes, some relative factors regarding maternal Hb at surgery (g/dL), presence of vaginal bleeding or uterine contraction at admission, emergency surgery, PASDs type of percreta, time duration from skin incision to fetal delivery (mins), gestational age (weeks), and birth weight (gram) are shown in Table 4. Using multivariable logistic regression, the study revealed that time duration from skin incision to fetal delivery (mins) and gestational age (weeks) were the independent factors more likely to be associated with a 5-min APGAR score less than of 7 points (adverse neonatal outcome). Concisely, one minute-reduced time duration from skin incision to fetal delivery increased the 5-min-APGAR score by 0.023 points and decreased the risk of adverse neonatal outcome by 2.2% with adjusted OR, 95% CI: 0.978 (0.962–0.993), *p* = 0.006. Meanwhile, one week-decreased gestational age reduced the 5-min-APGAR score by 0.684 points and increased nearly two fold odds of the adverse neonatal outcome with adjusted OR, 95% CI: 1.983 (1.600–2.456), *p* < 0.0001.

**Table 4:** Univariate and multivariable logistic regression for risk factors related to poor outcome of newborn after adjusting for gestational age and birthweight

Logistic regression		Univariate				Multivariable			
**Factors**		**B**	**S.E**	**Crude OR 95% CI**	***p*-value**	**B**	**S.E**	**Adjusted OR 95% CI**	***p*-value**
Maternal age (years)		0.021	0.021	1.021 0.980–1.063	0.369	-			
Number of CS scar (times)	≥ 2	0.282	0.214	1.326 0.872–2.017	0.188	-			
	< 2			Ref					
Parity (times)	≥ 2	0.178	0.203	1.195 0.802–1.779	0.381	-			
	< 2			Ref					
Pre-pregnancy BMI (kg/m^2^)		-0.032	0.037	0.969 0.901–1.042	0.393	-			
Surgical method	MOSCUS	0.079	0.242	1.082 0.674–1.737	0.745	-			
	CS hysterectomy			Ref					
Placental location	Anterior	0.141	0.203	1.152 0.774–1.714	0.487	-			
	Others			Ref					
Hb at surgery (g/dL)		**0.184**	**0.078**	**1.202 1.032–1.399**	**0.018**	0.031	0.093	1.031 0.860–1.238	0.739
Labor symptoms at admission	Vaginal bleeding/uterine contraction	**0.057**	**0.206**	**1.784 1.191–2.673**	**0.005**	0.148	0.239	1.159 0.725–1.853	0.537
	Asymptomatic			**Ref**				Ref	
Mode of surgery	Emergency	**0.776**	**0.274**	**2.173 1.271–3.714**	**0.005**	0.28	0.359	1.329 0.658–2.685	0.428
	Planned			**Ref**				Ref	
Type of PASDs	Percreta	**0.518**	**0.204**	**1.679 1.125–2.507**	**0.011**	0.394	0.234	1.484 0.939–2.344	0.0911
	Accreta-Increta			**Ref**				Ref	
Time duration from skin incision to fetal delivery (min)		**-0.022**	**0.007**	**0.978 0.965–0.992**	**0.002**	**-0.023**	**0.008**	**0.978 0.962–0.993**	**0.006**
Gestational age (weeks)		**0.729**	**0.090**	**2.073 1.738–2.472**	** < 0.0001**	**0.684**	**0.109**	**1.983 1.600–2.456**	** < 0.0001**
Newborn weight (gram)		**0.002**	**0.0001**	**1.002 1.001–1.002**	** < 0.0001**	0.0001	0.0001	1.000 1.000–1.001	0.495

*B* Beta-coefficient, *CI* Confidence interval, *CS* Cesarean section, *MOSCUS* the modified one-step conservative uterine surgery, *PSADs* Placenta accreta spectrum disorders, *OR* odds ratio, *S.E* standard error. 5-min APGAR score less than 7 points was considered for poor neonatal outcome

Statistically significant *p*-values (< 0 .05) are written in bold

## Discussions

In the present study, among pregnant women with placenta accreta spectrum disorders (PASDs), the rate of emergency cesarean section (CS) (65/468 cases) was lower than planned surgery (403/468 cases). Nearly 94% of cases in planned surgery were delivered after 34 weeks of gestation. This optimal timing of delivery is successfully expected since our center is a tertiary referral hospital where antenatal management plays an important role. Emergency cesarean surgery without careful pre-operative preparation leads to unexpected outcomes for both mother and infant including greater estimated blood loss, anemia after surgery, and requirement for blood transfusion as described in Table 1.

Although a multidisciplinary team has been established for all pregnant women with PASDs at our center, adverse neonatal outcomes occurred when the patients were operated in emergency conditions. This is almost related to premature delivery [27]. According to Salmanian et al., less than half of placenta accreta spectrum patients had scheduled delivery within the recommended gestational age of 34 0/7 to 35 6/7 weeks [28]. Iatrogenic preterm delivery causes potential neonatal implications, leading to a risk for significant respiratory morbidity [22]. Moreover, emergency management occurs usually in conditions with a lack of antenatal corticosteroids for fetal lung maturation and magnesium sulfate therapy for neuroprotection [29]. This result is in line with Nguyen et al. regarding neonatal outcomes, which included 95 cases in the emergency cesarean delivery group and 160 cases in the planned delivery group. The planned delivery is strongly associated with better neonatal outcomes compared to emergency cesarean delivery [30].

Although the cut-off of the APGAR score at 1 min less than 3 pts is required for neonatal intervention, some documents have demonstrated that the APGAR score at 1 min is not related to the adverse long-term outcomes, even at 1 pt or 3 pts [31]. Regarding the 5-min APGAR score, a total score of fewer than 7 pts relates significantly to poor neonatal outcomes and increases tenfold NICU admission despite non-PASDs cesarean delivery [32–34]. In our study, using the APGAR score of less than 7 pts as the adverse neonatal outcome, the study found that the neonatal requirement of intervention after birth, NICU admission, and the length of stay (LOS) at NICU admission of more than 7 days were significantly greater in emergency group versus planned surgery. In the report of Morlando et al., regarding the gestational age of 24–34 weeks, the NICU LOS in emergency surgery lasted significantly longer than in planned surgery, 12 (0–31) days vs. 0 (0–6) days, respectively, *p* < 0.001 [35].

On the contrary, the neonatal outcomes were not significantly different between the MOSCUS and the Cesarean hysterectomy in our study. This result was explained by the planned surgery performed in almost all cases and the PASDs pregnant women were sufficiently assessed by prenatal care before surgical intervention. Conversely, the worsening issue was the time duration from skin incision to fetal delivery with anesthetic drug exposure [36]. According to the case–control study of Munoz et al. on 107 PASD cases and 76 cases of placenta previa with prior cesarean section, neonates born to the PASDs mother had higher rates of respiratory morbidity, and general anesthesia is a significant contributor to respiratory outcomes [37]. However, the time duration from skin incision to fetal delivery (min) was similar in both surgical methods (Table 3 and Supplementary Table 1). In accordance with the finding of Placios-Jaraquemada et al., who concluded that conservative surgery in pregnancies complicated by PASDs does not seem to increase the risk of adverse neonatal outcomes. However, the grade of severe invasion was also related to the earlier termination of gestational age, lower birth rates, and severe outcomes due to preterm birth [38].

Recently, Morlando et al. suggested that in the context of adequate expertise and available resources, deferring delivery in women with no significant antenatal bleeding and no risk factors for preterm birth until more than 36 weeks of gestation can be considered to improve fetal outcomes [35]. Nevertheless, this management should be carefully considered because of the risk of severe placental invasion for disastrous maternal outcomes. The later gestational age of pregnancy results in a deeper invasion of placenta and poorer outcomes [39]. Thus, the optimal timing of delivery should be carefully assessed according to the practical guidelines to minimize the unexpected consequences [40].

After multilogistic conditional regression analysis, our study found two main factors the factors associated with increased risk of adverse neonatal outcomes including the early gestational age and time from skin incision to fetal extraction. Regarding gestation age, the timing of planned delivery in PASDs remains a challenge for all obstetricians worldwide. Approximate half of cases tend to undergo spontaneous labor before the gestational-age expectation. According to the finding of Kasraeian et al., eighty-eight out of 198 neonatal cases (44.4%) were sent to NICU due to prematurity. The gestational age from 34 to 37 weeks occupied up to 46% [41]. In our study, we found that the gestational age at birth was significantly related to the APGAR score of less than 7 pts. This result was similar to the report of Oğlak et al. [42]. Meanwhile, the rate of infants experiencing any major neonatal morbidity was decreasing from 25% at 34 + 1 to 36 + 0 weeks to 19% at > 36 + 0 weeks [43].

In cesarean delivery without PASDs, general anesthesia is also a risk for fetal health conditions.On the other hand, the anterior location of placenta previa could cause difficulty in extracting the fetus while the amniotic membrane was ruptured and the amniotic fluid mixed with the bloodstream. Particularly, the presentation of the fetus was transverse lie or breech, instead of cephalic presentation (Supplementary Video 3, 4 and 5). In our experience, as concerning the newborn outcomes, the neglect of Step 1 (proliferative vascular ligation before uterine incision) causes uncontrolled massive bleeding after fetal delivery and consequently leads to maternal mortality. Thus, a rapidly careful performance by expert surgeons in PASDs centers is necessary regarding these cases.

### Strength and limitations

One the strength of this study was carried out at a tertiary referral hospital with a large number of cases of PASDs in a duration period of one year, whereas the various studies had numerous PASDs cases from the multicenter or required a long-lasted period of study. Our study identified the diagnosis of PASDs at the time of cesarean section and confirmed the diagnosis by histological endpoint. Other studies assessed the PASDs by ultrasound without intraoperative diagnosis or lack of histology. Moreover, all infants in our study were equally assessed by multidisciplinary management according to the hospital protocol.

However, this was a retrospective study, thus the study could not avoid bias. This limitation might have originated from the study population, the fetuses might receive and might not receive prenatal corticosteroid therapy for fetal lung maturity. This was similar to magnesium sulfate for neuroprotection. These different conditions may be attributable to the neonatal outcomes. The study did not analyze the data for the group of fetal growth restriction (FGR) which might result in low APGAR score. The study missed data on umbilical cord arterial blood cord pH and gases, optimal parameters predicting neonatal outcome. The study described insufficiently all the results of laboratory tests during NICU admission and without follow-up after transferring hospital. The neonatal comorbidities such as intraventricular hemorrhage, necrotizing enterocolitis, severe infection, and meningitis were not counted separately. Due to the ethical issue of human subjects, the study could not conduct a prospective randomized controlled trial that assigned the pregnant women of PASDs to either the MOSCUS group or the Cesarean hysterectomy group, and the sample size of the study was not calculated for the study design. In addition, the study followed the timing of delivery in PASDs without dividing the neonates into subgroups according to the diverse categorization of PASDs type based on gestational age for comparison.

### Study implications and future research

This study added to the literature the neonatal outcome in types of surgery (emergency CS versus planned CS) with a large number of PASDs pregnancies under general anesthesia. In addition, the study showed no significant difference between the MOSCUS method and Cesarean hysterectomy. The study also found the independent factors related to the adverse neonatal outcome including time duration from skin incision to fetal delivery (min) and gestational age (weeks). Thus, clinicians should manage strictly the PASDs at tertiary referral hospitals to avoid preterm emergency cesarean section and the practitioner’s skill hand needs to be enhanced to perform rapidly the Step 1 (vesicouterine dissection and proliferative vascular ligation) to shorten the time duration from skin incision to fetal delivery in the MOSCUS method.

However, this study might not reflect the condition in the whole country since the surgical techniques remain different. A prospective propensity-matched-score multicenter study through the telemedicine strategy in PASDs management is necessary in southern Vietnam. The future research may be generalizable to centers with well-trained multidisciplinary teams of surgeons, anesthetists, and neonatologists. By experienced surgeons with the same hand-skill, the team could assess the different outcomes of the newborn in less than 30-min group and more than 30-min group when the surgeon performs the Step 1 before fetal delivery. We also could investigate the neonatal outcomes in different subgroups of gestational age at 28–34 weeks of GA and 34–36 weeks of GA regarding both singleton and twin pregnancies. Other predicting tools could be added to evaluate accurately the cerebral injuries of the newborn besides the APGAR score. Moreover, the long-term infant outcome under general anesthesia needed to be considered since the PASDs required longer time duration from skin incision to fetal delivery than that in cesarean section of uncomplicated pregnancies.

## Conclusions

In summary, the management of surgical methods including the MOSCUS method and Cesarean hysterectomy did not contribute significantly to the risk of adverse neonatal outcomes, whereas the increased rate of neonatal complication is related to emergency cesarean section. Vaginal bleeding with frequent uterine contraction and hemoglobin level might be suggestive factors relating to emergency cesarean surgery. The time duration from the skin incision to fetal delivery and gestational age should be noted in the surgery of placenta accreta spectrum disorders. Additionally, after balancing the risks and the benefits for mother and newborn, proper prenatal management of placenta accreta spectrum disorders is strictly required to reduce the emergency preterm cesarean delivery before the recommended delivery timing window of PASDs. Further data is required to strengthen these findings and evaluate the long-term neonatal outcomes in PASDs surgery under general anesthesia at different centers.

### Supplementary Information

**Additional file 1: Supplementary Table 1. **Characteristics and outcomes of pregnancies underwent planned cesarean section from 34 weeks of gestation in the MOSCUS method and Cesarean hysterectomy group.

**Additional file 2: Supplementary Table 2.** Univariate and multivariable logistic regression for risk factors related to emergency cesarean delivery after adjusting for gestational age and birthweight.

**Additional file 3: Supplementary Figure 1. **Graphs show the comparison of the frequency of newborns with the Apgar score in type of surgery and surgical methods using independent samples Mann-Whitney U-test. A. Apgar score at 1 min in emergency cesarean surgery versus planned cesarean surgery. B. Apgar score at 5 mins in emergency cesarean surgery versus planned cesarean surgery. C. Apgar score at 1 min in Cesarean hysterectomy versus MOSCUS method. D. Apgar score at 5 mins in Cesarean hysterectomy versus MOSCUS method.

**Additional file 4: Supplementary video 1 and 2.** Videos shows the hemostatic procedures and vesicouterine dissection before fetal delivery in cesarean section with placenta accreta spectrum disorders (PASDs). This important step requires commonly more time than the cesarean section without PASDs.

**Additional file 5: Supplementary video 3,4, and 5.** Videos shows the difficulties in fetal extraction after uterine incision and amniotic membrane is ruptured. Anterior placental location of placenta accreta spectrum disorders (PASDs), a massive bleeding, an amniotic fluid, and mobile position of fetal presentation in preterm pregnancy are attributable factors causing the fetal extraction more difficult in cesarean section with PASDs.

## Data Availability

The datasets generated and/or analysed during the current study are not publicly available due to patient privacy and restrictions apply which was used under license of our hospital. However, data are available from the corresponding author on reasonable request and permissions of Tu Du Hospital, Vietnam.

## Abbreviations

ACOG: American College of Obstetricians and Gynecologists
CS: Cesarean section
LMICs: Low-middle income countries
MOSCUS: Modified One-Step Conservative Uterine Surgery
PASDs: Placenta accreta spectrum disorders
FIGO: International Federation of Gynecology and Obstetrics
