# Supplementary material for: Neonatal outcomes in the surgical management of placenta accreta spectrum disorders: a retrospective single-center observational study from 468 Vietnamese pregnancies beyond 28 weeks of gestation
Source: BMC Pregnancy Childbirth. 2024 Apr 2;24:228. doi: 10.1186/s12884-024-06349-7 (PMC10986094; doi:10.1186/s12884-024-06349-7)
Supplement: Supplementary file 1 — Additional file 1: Supplementary Table 1. Characteristics and outcomes of pregnancies underwent planned cesarean section from 34 weeks of gestation in the MOSCUS method and Cesarean hysterectomy group. [file 12884_2024_6349_MOESM1_ESM.docx]

**Supplementary Table 1.** Characteristics and outcomes of pregnancies underwent planned cesarean section from 34 weeks of gestation in the MOSCUS method and Cesarean hysterectomy group

| **Characteristics and outcomes** | | | | **MOSCUS**  **N = 176** | **Cesarean**  **Hysterectomy**  **N = 64** | **Total**  **N = 240** | ***p-*value** |
| --- | --- | --- | --- | --- | --- | --- | --- |
| **Maternal age (years)** | mean ± SD  (min-max) | | | 33.16 ± 4.88 | 33.97 ± 4.90 | 33.38 ± 4.89  (18-49) | 0.260^┼^ |
| **Maternal BMI**  **(kg/m^2^)** | mean ± SD  (min-max) | | | 22.01 ± 2.74 | 22.99 ± 2.92 | 22.27 ± 2.82  (16.42-33.29) | 0.018^┼^ |
| **Parity**  **(times)** | mean ± SD  (min-max) | | | 1.63 ± 0.74 | 1.84 ± 0.78 | 1.69 ± 0.75  (0-5) | 0.052^┼^ |
|  | ≤ 1 | | | 86 (48.9) | 21 (32.8) | 107 (44.6) | 0.027^*^ |
|  | ≥ 2 | | | 90 (51.1) | 43 (67.2) | 133 (55.4) |  |
| **Cesarean scar**  **(times)** | mean ± SD  (min-max) | | | 1.35 ± 0.62 | 1.61 ± 0.66 | 1.42 ± 0.64  (0-3) | **0.005^┼^** |
|  | ≤ 1 | | | 115 (65.3) | 29 (45.3) | 144 (60.0) | **0.005^**^** |
|  | ≥ 2 | | | 61 (34.7) | 35 (54.7) | 96 (40.0) |  |
| **Hb at admission**  **(g/dL)** | mean ± SD  (min-max) | | | 11.40 ± 1.19 | 10.99 ± 1.53 | 11.29 ± 1.30  (6.8-14.4) | **0.030^┼^** |
| **Hb at pre-surgery**  **(g/dL)** | mean ± SD  (min-max) | | | 11.42 ± 1.21 | 11.20 ± 1.29 | 11.36 ± 1.17  (7.0-14.4) | 0.198^┼^ |
| **Hb at discharge**  **(g/dL)** | mean ± SD  (min-max) | | | 9.30 ± 1.55 | 8.96 ± 1.45 | 9.21 ± 1.53  (6.1-14.4) | 0.126^┼^ |
| **Type of intraoperative PASDs** | Accreta | | | 6  (3.4) | 1  (1.6) | 7  (2.9) | **< 0.0001^*^** |
|  | Increta | | | 61  (34.7) | 2  (3.1) | 63  (26.3) |  |
|  | Percreta | | | 109  (61.9) | 61  (95.3) | 170 (70.8) |  |
| **Estimated blood loss (ml)** | Median  IQR  [Q1-Q3]  (min-max) | | | 1000  [600-1800]  (200-6000) | 1500  [800-2500]  (300-6500) | 1200  [600-2000]  (200-6500) | **0.0001^┼┼^** |
| **Operative time duration (mins)** | mean ± SD  (min-max) | | | 151.36 ± 41.73 | 187.03 ± 56.81 | 160.88 ± 48.74  (60-365) | **0.0001^┼^** |
| **Time from skin incision to fetal delivery (mins)** | mean ± SD  (min-max) | | | 30.23 ± 14.76 | 38.33 ± 20.71 | 32.07 ± 16.60  (5-95) | **0.022^┼^** |
| **Intraoperative complication** | Absence | | | 169 (96.0) | 49 (76.6) | 218 (90.8) | **<**  **0.0001^**^** |
|  | Presence | | Bladder perforation | 6 (3.4) | 9 (14.1) | 15 (6.3) |  |
|  |  |  | Ureter injury | 0 (0.0) | 3 (4.7) | 3 (1.3) |  |
|  |  |  | Rectal/bowel perforation | 0 (0.0) | 3 (4.7) | 3 (1.3) |  |
|  |  |  | Large vessel injury | 1 (0.6) | 0 (0.0) | 1 (0.4) |  |
| **Hospital length of stay (days)** | mean ± SD  (min-max) | | | 7.06 ± 2.12 | 7.16 ± 2.64 | 7.08 ± 2.26  (2-20) | 0.764^┼^ |
| **Blood transfusion** | No | | | 161 (91.5) | 63 (98.4) | 224 (93.3) | **0.002^*^** |
|  | Yes | | | 15 (8.5) | 1 (1.6) | 16 (6.7) |  |
| **Postpartum hemorrhage** | No | | | 175 (99.4) | 62 (96.9) | 237 (98.8) | 0.174^**^ |
|  | Yes | | | 1 (0.6) | 2 (3.1) | 3 (1.3) |  |
| **Postoperative infection** | No | | | 161 (91.5) | 63 (98.4) | 224 (93.3) | **0.077^**^** |
|  | Yes | | | 15 (8.5) | 1 (1.6) | 16 (6.7) |  |
| **Gestational age**  **(weeks)** | mean ± SD  (min-max) | | | 35.83 ± 1.36 | 35.42 ± 1.45 | 35.72 ± 1.39  (34.0-40.5) | **0.048^┼^** |
| **Birth weight**  **(gram)** | mean ± SD  (min-max) | | | 2647.50 ± 442.58 | 2626.70 ± 393.65 | 2641.95±429.37  (1650-4100) | 0.741^┼^ |
| **APGAR score at 1 min (pts)** | median  IQR [1-3]  (min-max) | | | 5  [5-6]  (1-7) | 5  [5-6]  (1-7) | 5  [5-6]  (1-7) | 0.682^┼┼^ |
|  | ≤ 3 | | | 7  (4.0) | 2  (3.2) | 9  (3.8) | 1.000^**^ |
|  | >3 | | | 169  (96.0) | 61  (96.8) | 230  (96.2) |  |
| **APGAR score at 5 min (pts)** | median  IQR [1-3]  (min-max) | | | 7  [6-7]  (4-8) | 7  [6-7]  (3-8) | 7  [6-7]  (3-8) | 0.935^┼┼^ |
|  | <7 | | | 51  (29.0) | 19  (30.2) | 70  (29.3) | 0.860^*^ |
|  | ≥7 | | | 125  (71.0) | 44  (69.8) | 169  (70.7) |  |
| **Need of oxygen support** | No | | | 90  (51.1) | 37  (57.8) | 127  (52.9) | 0.360^*^ |
|  | Yes | | | 86  (48.9) | 27  (42.4) | 113  (47.1) |  |
|  | Type of intervention | Cannula/mask | | 65  (36.9) | 20  (31.3) | 85  (35.4) |  |
|  |  | CPAP | | 11  (6.3) | 3  (4.7) | 14  (5.8) |  |
|  |  | Mechanical ventilation^a^ | | 10  (5.7) | 4  (6.3) | 14  (5.8) |  |
| **NICU admission** | No | | | 110  (62.5) | 25  (39.1) | 91  (37.9) | 0.825^*^ |
|  | Yes | | | 66  (37.5) | 39  (60.9) | 149  (62.1) |  |
| **Length of stay at NICU (day)** | median  IQR [1-3]  (min-max) | | | 6.5  [5-9]  (2-34) | 6  [4-9]  (1-21) | 6  [5-9]  (1-34) | 0.778^┼┼^ |
|  | ≥ 7 | | | 23  (34.8) | 12  (48.0) | 35  (38.5) | 0.250^*^ |
|  | <7 | | | 43  (65.2) | 13  (52.0) | 56  (61.5) |  |
| **Major neonatal outcome** | Alive | | | 176  (100.0) | 62  (96.9) | 238  (99.2) | 0.070^**^ |
|  | Death/transferred hospital | | | 0  (0.0) | 2  (3.1) | 2^b^  (0.8) |  |

*^a^: Mechanical ventilation including invasive and non-invasive method, ^b^in-utero fetal death (n=1), transferred hospital due to the respiratory distress syndrome (n=1). Missing data was calculated for in-utero fetal death before cesarean section under general anesthesia.*

** Chi-square test ^**^ Fisher’s exact test ^┼^Independent sample-t test, ^┼┼^ Independent sample Mann Whitney test, asymptotic sig (2-sided) of p-value. IQR [1-3]: interquartile range [25%-75%]*

*CPAP: continuous positive airway pressure, NICU: neonatal intensive care unit, Hb: hemoglobin.*
