# Supplementary material for: Neonatal outcomes in the surgical management of placenta accreta spectrum disorders: a retrospective single-center observational study from 468 Vietnamese pregnancies beyond 28 weeks of gestation
Source: BMC Pregnancy Childbirth. 2024 Apr 2;24:228. doi: 10.1186/s12884-024-06349-7 (PMC10986094; doi:10.1186/s12884-024-06349-7)
Supplement: Supplementary file 2 — Additional file 2: Supplementary Table 2. Univariate and multivariable logistic regression for risk factors related to emergency cesarean delivery after adjusting for gestational age and birthweight. [file 12884_2024_6349_MOESM2_ESM.docx]

**Supplementary Table 2. Univariate and multivariable logistic regression for risk factors related to emergency cesarean delivery after adjusting for gestational age and birthweight**

| **Logistic regression** | | **Univariate** | | | | **Multivariable** | | | |
| --- | --- | --- | --- | --- | --- | --- | --- | --- | --- |
| **Factors** |  | **B** | **S.E** | **Crude OR**  **95% CI** | ***p*-value** | **B** | **S.E** | **Adjusted OR**  **95% CI** | ***p*-value** |
| Number of CS scar  (times) | ≥ 2 | 0.341 | 0.277 | 1.406  0.817-2.419 | 0.218 | - | | | |
|  | < 2 |  |  | Ref |  |  |  |  |  |
| Parity  (times) | ≥ 2 | 0.358 | 0.268 | 1.431  0.846-2.412 | 0.182 | - | | | |
|  | < 2 |  |  | Ref |  |  |  |  |  |
| Type of PASDs | Percreta | 0.204 | 0.270 | 1.227  0.723-2.081 | 0.449 | - | | | |
|  | Accreta-increta |  |  | Ref |  |  |  |  |  |
| Labor symptoms at admission | Vaginal bleeding/uterine contraction | **2.662** | **0.375** | **14.332**  **6.870-29.895** | **<0.0001** | **2.526** | **0.386** | **12.502**  **5.868-26.640** | **<0.0001** |
|  | Asymptomatic |  |  | **Ref** |  |  |  | **Ref** |  |
| Pre-pregnancy BMI (kg/m^2^) | | **0.140** | **0.055** | **1.150**  **1.034-1.280** | **0.010** | 0.106 | 0.057 | 1.112  0.995-1.243 | 0.060 |
| Hb at admission  (g/dL) | | **0.330** | **0.094** | **1.391**  **1.156-1.673** | **< 0.0001** | **0.259** | **0.098** | **1.295**  **1.069-1.569** | **0.008** |

*B:Beta-coefficient, BMI: Body mass index, CI: Confidence interval, CS: Cesarean section, OR: odds ratio, PSADs: Placenta accreta spectrum disorders, Hb: Hemoglobin, S.E: standard error.*
