# Supplementary figures and images for: Neonatal outcomes in the surgical management of placenta accreta spectrum disorders: a retrospective single-center observational study from 468 Vietnamese pregnancies beyond 28 weeks of gestation
Source: BMC Pregnancy Childbirth. 2024 Apr 2;24:228. doi: 10.1186/s12884-024-06349-7 (PMC10986094; doi:10.1186/s12884-024-06349-7)

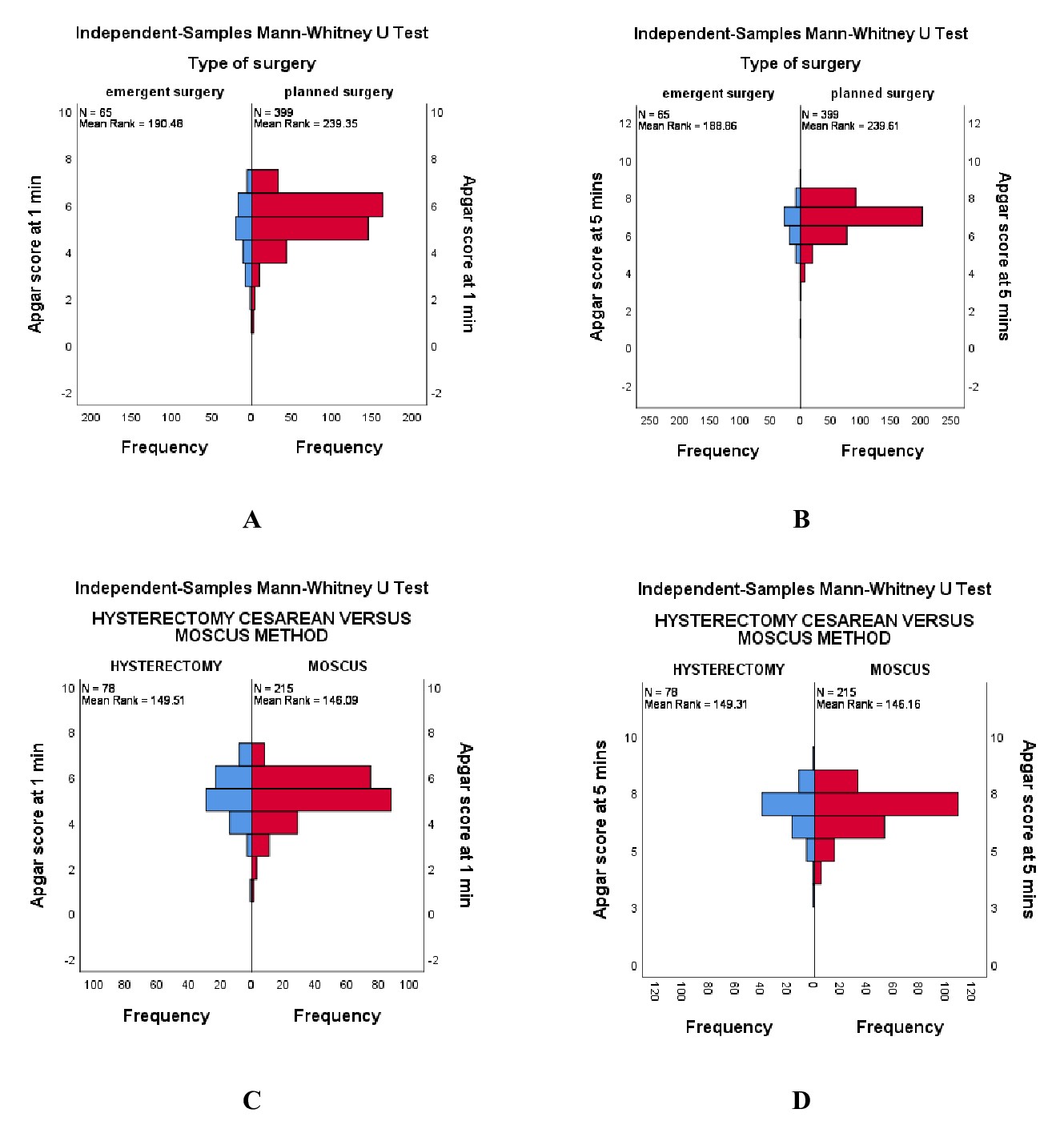

Supplement: Supplementary file 3 — Additional file 3: Supplementary Figure 1. Graphs show the comparison of the frequency of newborns with the Apgar score in type of surgery and surgical methods using independent samples Mann-Whitney U-test. A. Apgar score at 1 min in emergency cesarean surgery versus planned cesarean surgery. B. Apgar score at 5 mins in emergency cesarean surgery versus planned cesarean surgery. C. Apgar score at 1 min in Cesarean hysterectomy versus MOSCUS method. D. Apgar score at 5 mins in Cesarean hysterectomy versus MOSCUS method. [file 12884_2024_6349_MOESM3_ESM.jpg]
